# Gait speed and multi-segment foot joint kinematics throughout the entire stance phase using statistical parametric mapping

**DOI:** 10.7717/peerj.21787

**Published:** 2026-10-21

**Authors:** Hwigeum Jeong, Joseph Hamill, Katherine A. Boyer, Richard E.A. van Emmerik

**Affiliations:** 1 Department of Kinesiology, University of Massachusetts at Amherst, Amherst, MA, United States of America; 2 Department of Human Movement Studies, University of Ostrava, Ostrava, Czech Republic

**Keywords:** Joint kinematics, Multi-segment foot model, Walking speed, Statistical parametric mapping

## Abstract

The foot is a multi-segment structure whose segments function together. Due to its ongoing connection with the support surface, the foot must be capable of managing loads that vary with gait speed. Previous studies have provided considerable insight into the relationship between foot joint kinematics and gait speed. However, their primary reliance on discrete measures, such as peak joint angles, has limited understanding of how these relationships evolve throughout the stance phase. The aim of this study was to investigate the effects of gait speed on full time-series trajectories of multiplanar foot joint kinematics using statistical parametric mapping. According to our findings, faster walking primarily increased ankle eversion and midfoot dorsiflexion during weight acceptance (∼17–29% of the stance phase). In contrast, during propulsion, faster walking increased ankle plantar flexion (∼80–100% of the stance phase) and inversion (∼82–90%), as well as midfoot plantar flexion (∼82–100%) and adduction (∼90–98%), together with greater dorsiflexion of the first metatarsophalangeal joint after heel-off (∼43–98% of the stance phase). These multiplanar joint kinematic alterations across the ankle–foot joints throughout the stance phase reflect greater foot-arch collapse in early stance and foot-arch recoil in late stance, which may facilitate appropriate energy absorption and generation in response to increasing gait speed. Importantly, these adaptive foot motions were not captured by previous discrete analyses, which may obscure phase-dependent changes during gait. The present continuous time-series approach revealed detailed temporal adaptations in foot kinematics across the stance phase, providing a more comprehensive characterization of gait mechanics.

## Introduction

In human locomotion the foot is the body segment making continuous contact with the ground, playing a critical role in shock attenuation (Whittle, 1999), energy transmission (Ker et al., 1987), and postural control (Wong et al., 2008; Buldt et al., 2018). Unlike the traditional view of the foot as a single fused segment, it is a complex assembly of multiple small segments that articulate with one another to produce integrated motions essential for foot function. For example, during the weight-acceptance phase, the medial longitudinal arch—the foot’s most prominent arch—temporarily collapses, whereas during the propulsion phase it elevates again due to its elastic recoil (Huson, 1991; Hicks, 1954). These motions arise from the coordinated actions of the multi-segment structure across all three planes of motion. Specifically, the arch collapse is driven by the coordinated motions of supination–adduction–flexion at the ankle joint, concomitant midtarsal supination, and pronation twist of the forefoot (Hicks, 1953). In contrast, elevation of the arch results from the reciprocal coupling patterns across these ankle–foot joints (Hicks, 1953).

Due to its many degrees of freedom, the foot can adapt to environmental changes, enabling humans not only to walk on different surfaces (*e.g.*, flat or uneven terrain) but also to adjust to varying loads by deforming the longitudinal arch (Ker et al., 1987; Caravaggi et al., 2010). This adaptability highlights the close relationship between foot function and gait speed. Gait speed has been shown to influence multiplanar ankle–foot kinematics, affecting the ankle and midfoot joints (Van Hoeve et al., 2017; Tulchin et al., 2009; Arnold et al., 2014), as well as the first metatarsophalangeal (MTP) joint (Dubbeldam et al., 2010; Sun et al., 2018), which functions as a stiff lever that modulates foot stiffness through the windlass mechanism, wherein hallux dorsiflexion tensions the plantar aponeurosis (Hicks, 1954). However, the results from previous studies are not consistent. For instance, whereas Dubbeldam et al. (2010) reported greater peak hallux dorsiflexion at faster gait speeds, Sun et al. (2018) observed greater dorsiflexion at slower speeds. Comparing these findings is challenging because discrete metrics do not account for temporal characteristics. Particularly, the timing of peak joint angles potentially differs across individuals and between trials, which hinders direct comparisons across gait cycles. Gait speed influences not only the magnitude of joint motion but also its timing across the entire gait cycle as shown by joint angle curves (Tulchin et al., 2009; Sun et al., 2018), thereby systematically shaping foot-arch dynamics throughout the stance phase (Caravaggi et al., 2010). Consequently, it is crucial to investigate the multiplanar joint kinematics of the foot over the entire stance phase to better understand how its multi-segment structure re-organizes its dynamic coordination to adapt to changes in gait speed.

In time series analyses, reliance on discrete metrics, such as peak joint angles, can introduce bias. Pataky, Robinson & Vanrenterghem (2013) illustrated this using example data (see the Appendix in Pataky, Robinson & Vanrenterghem, 2013). Because current states in biological systems are not necessarily independent of their history (Haken, 1977), joint kinematic signals in the foot, regarded as a dynamic system, are likely time dependent. To capture effects of gait speed, a control parameter that changes macroscopic patterns across the gait cycle (Van Emmerik, Miller & Hamill, 2014), statistical parametric mapping (SPM) is a promising analytical tool. SPM effectively controls family-wise type I error based on random field theory, which accounts for the spatial or temporal dependence among adjacent observations in a continuous statistical field (Pataky, Robinson & Vanrenterghem, 2013). In this continuous random field, SPM provides temporal information that identifies when statistical differences occur, while minimizing violations of the family-wise type I error rate. SPM has been applied to investigate foot joint kinematics in clinical populations across the entire gait cycle (Stevens et al., 2022) and to examine foot joint kinetics across different gait speeds throughout the stance phase (Eerdekens, Deschamps & Staes, 2019). However, to the best of our knowledge, little is known about how gait speed affects foot joint kinematics over the stance phase.

Therefore, the aim of this study was to investigate the effects of gait speed on the joint kinematics of the multi-segment foot throughout the entire stance phase. Multiplanar joint angles of the ankle, midfoot, and first MTP joints were compared across slow, preferred, and fast walking speed conditions using a one-way repeated measures analysis of variance (ANOVA) with SPM. Faster walking has been shown to increase foot arch collapse during the weight acceptance phase due to increased compressive forces, while further increasing foot arch height during propulsion to meet mechanical demands (Caravaggi et al., 2010). Based on this biomechanical reasoning, we hypothesized that faster walking would increase ankle pronation–abduction–dorsiflexion and midfoot pronation during weight acceptance, whereas it would increase ankle supination–adduction–plantar flexion and midfoot supination during propulsion (Hicks, 1953). In addition, we hypothesized that faster walking would increase first MTP joint dorsiflexion during late stance, consistent with the windlass mechanism (Hicks, 1954).

## Method

### Participants

This cross-sectional study recruited 12 volunteers (six men and six women, age: 24 ± 3.8 years; weight: 69.37 ± 19.75 kg; height: 1.73 ± 0.12 m). All participants were screened *via* an online questionnaire (Qualtrics, UT, USA) and were required to report no apparent foot malalignment (including low-arched foot or high-arched foot), no signs of foot hypo- and hyper-mobility, and no history of cardiopulmonary, musculoskeletal, or other conditions affecting gait. Individuals were excluded if they reported any foot deformities, were younger than 18 or older than 35 years, or had experienced musculoskeletal injuries within the past 6 months. All participants provided written informed consent prior to participation. All procedures were approved by the Institutional Review Board of the University of Massachusetts Amherst (Approval No. 3558).

### Protocol

All participants wore a spandex top and short sport pants. Once height and weight were obtained, all participants were given sufficient time to familiarize themselves with walking barefoot on a treadmill before beginning the experiments. Preferred walking speed (PWS) was determined based on the method of Jordan, Challis & Newell (2007). The initial speed of the treadmill was relatively slow and was increased by 0.1 ms^−1^ every 5–10 s until the participants reported that they were walking at their PWS. Then, the speed of the treadmill was increased to 1.5 ms^−1^ and decreased by 0.1 ms^−1^ every 5–10 s until a PWS was re-established. This procedure was repeated until the deviation of PWS was less than 0.2 ms^−1^. Then, after a static trial to define a biomechanical model on a global coordinate system, the participants walked on the treadmill for five minutes at the slow gait speed (SWS = 70% of PWS), the preferred gait speed (100% of PWS), and the fast gait speed (FWS = 130% of PWS). Gait speed conditions were randomized across participants. There was at least a 3-minute interval between trials to avoid the effects of fatigue.

### Experimental set-up

A motion capture system (Qualisys, Inc., Gothenburg, Sweden) consisting of eight cameras was used to track the movement of 19 body segments (see Appendix A for marker setting) with a sampling rate of 200 Hz. The reflective markers were smoothed with a dual pass 4th order, low-pass Butterworth filter at 6 Hz prior to creating a multi-segment foot model in Visual 3D™ (C-Motion Inc., Germantown, MD, USA). A multi-segment foot model developed by Bruening, Cooney & Buczek (2012) was used due to its high inter- and intra-rater reliability. This foot model consists of the shank, hindfoot, forefoot, and hallux theoretically assigned with six degrees of freedom—rotational and translational movement in the three orthogonal planes—except for the hallux that moves in two planes, sagittal and transverse (Bruening, Cooney & Buczek, 2012). All tracking markers were retained for both the static calibration trial and dynamic trials, but the following markers for segment reference frames were removed after the static trial due to potential interruption of walking: markers on the medial epicondyle of the femur, medial malleolus of the ankle, medial calcaneus, base of first metatarsus, and navicular. Detailed marker placements are provided in the supplementary figure (Appendix B) for readers seeking additional information. Based on this, we selected the lateral calcaneus marker to track the hindfoot segment, instead of the medial calcaneus marker, unlike Bruening, Cooney & Buczek (2012). Joint kinematics were defined by the distal segment moving based on the proximal segment using a Cardan Xyz sequence of rotations (Bruening, Cooney & Buczek, 2012). The ankle joint angle represents the relative motion of the hindfoot with respect to the shank; the midfoot joint angle is the relative motion of the forefoot on the hindfoot; and the first MTP angle is the relative motion of the hallux on the forefoot.

### Data analysis

Joint angles were obtained in the sagittal (flexion/extension), frontal (inversion/eversion) and transverse planes (internal rotation/external rotation or adduction/abduction), except for the hallux angles, which were measured only in the sagittal and transverse planes, based on the degrees of freedom of the first MTP joint (Gómez et al., 2023). The stance phase was divided in different phases based on a previous study (Stevens et al., 2022): loading response (0%–20%), mid-stance (20%–50%), terminal stance (50%–80%), and pre-swing (80%–100%).

### Statistical analysis

Joint angles were time normalized over the stance phase (0–100%). The within-subject averages of the time-normalized joint angles over the 180 cycles of right footsteps were obtained for each percent stance in each gait speed condition. For statistical analyses of the continuous kinematic curve, a one-way repeated-measures ANOVA (*α* = 0.05) was performed using SPM (https://spm1d.org/; version M.0.4.10). If a main effect was observed, *post hoc* paired SPM *t*-tests with Bonferroni correction (adjusted critical value = 0.05/3) were conducted. Mean Cohen’s d effect sizes across regions of significant differences were calculated and interpreted as small (0.2), medium (0.5), and large (0.8), according to Cohen (2013).

## Results

The mean SWS, PWS, and FWS across the twelve participants were 0.79 ± 0.074 m⋅s^−1^, 1.13 ± 0.11 m⋅s^−1^, and 1.46 ± 0.14 m⋅s^−1^, respectively. SPM revealed significant main effects of gait speed on all joints across all planes. Table 1 summarizes the overall ANOVA results and Table 2 providing *post hoc* pairwise comparisons including the specific time intervals during which differences occurred.

### The ankle joint

In the sagittal plane, participants exhibited greater plantar flexion in FWS compared to PWS (*p* < 0.001; *d* = 0.77) and SWS (*p* < 0.001; *d* = 1.31) during pre-swing, whereas PWS showed greater plantar flexion than SWS (*p* = 0.006; *d* = 1.13) during the same phase (Table 2, top row; Fig. 1, top). In the frontal plane, ankle eversion was greater in FWS than in SWS during mid-stance (*p* < 0.001; *d* = 0.76), and ankle inversion was greater in FWS (*p* = 0.001; *d* = 0.97) and PWS (*p* = 0.001; *d* = 0.48) than in SWS during pre-swing, with no significant difference between FWS and PWS (Table 2, top row; Fig. 1, middle). In the transverse plane, both FWS (*p* < 0.001; *d* = 0.66) and PWS (*p* < 0.001; *d* = 0.41) showed greater internal rotation than SWS during terminal stance, while FWS and PWS did not differ significantly (Table 2, top row; Fig. 1, bottom).

### The midfoot joint

In the sagittal plane, midfoot plantar flexion was less in FWS than in PWS (*p* = 0.016; *d* = 1.72) and SWS (*p* = 0.016; *d* = 1.55) during loading response, and less in FWS than in SWS during mid-stance (*p* < 0.001; *d* = 0.80). However, FWS showed greater plantar flexion than PWS (*p* < 0.001; *d* = 0.64) and SWS (*p* = 0.001; *d* = 1.14) during pre-swing. PWS showed less plantar flexion than SWS during mid-stance (*p* < 0.001; *d* = 0.33) but greater plantar flexion during pre-swing (*p* = 0.014; *d* = 0.57) (Table 2, middle row; Fig. 2, top). In the frontal plane, midfoot eversion was greater in FWS than in PWS (*p* = 0.008; *d* = 0.34) and SWS (*p* = 0.007; *d* = 0.59) during pre-swing, with no difference between PWS and SWS (Table 2, middle row; Fig. 2, middle). In the transverse plane, FWS and PWS showed greater midfoot abduction than SWS during terminal stance (*p* = 0.007; *d* = 0.61 and *p* = 0.003; *d* = 0.34, respectively), and FWS exhibited greater midfoot adduction than SWS during pre-swing (*p* < 0.001; *d* = 1.26), while FWS and PWS did not differ significantly (Table 2, middle row; Fig. 2, bottom).

**Table 1:** Main effects observed in one-way repeated measures ANOVA using SPM.

Variables	Sagittal plane	Frontal plane	Transverse plane
The Ankle Joint	*P* = 0.001 (33%–58%)	*P* < 0.001 (14%–39%)	*P* < 0.001 (54%–95%)
	*P* < 0.001 (72%–100%)	*P* < 0.001 (71%–95%)	
The Midfoot Joint	*P* < 0.001 (0%–62%)	*P* = 0.049 (6%–8%)	*P* < 0.001 (22%–66%)
	*P* = 0.012 (82%–100%)	*P* = 0.030 (14%–25%)	*P* = 0.001 (77%–100%)
		*P* = 0.017 (85%–100%)	
The 1^st^ MTP Joint	*P* < 0.001 (33%–97%)	N/A	*P* = 0.050 (0%–2%)
			*P* < 0.001 (18%–53%)
			*P* = 0.003 (74%–100%)

**Notes.**

Loading response = 0–20%; Mid-stance = 20%–50%; Terminal stance = 50%–80%; and Pre-swing = 80%–100%.

**Table 2:** Results of Bonferroni *post hoc* tests using SPM.

		FWS *vs.* PWS	FWS *vs.* SWS	PWS *vs.* SWS
Ankle Joint	Sagittal Plane	*P* < 0.001 (85.4–98.0%) *d* = 0.77	*P* < 0.001 (78.4–100.0%) *d* = 1.31	*P* = 0.006 (94.5–100.0%) *d* = 1.13
	Frontal Plane	None	*P* < 0.001 (17.5–29.0%) *d* = 0.76	*P* = 0.001 (82.0–89.3%) *d* = 0.48
			*P* = 0.001 (82.6–91.0%) *d* = 0.97	
	Transverse Plane	None	*P* < 0.001 (58.6–82.4%) *d* = 0.66	*P* < 0.001 (58.8–83.6%) *d* = 0.41
Midfoot Joint	Sagittal Plane	*P* = 0.016 (0 –1.9%) *d* = 1.72	*P* = 0.016 (0.0–1.5%) *d* = 1.55	*P* < 0.001 (21.8–60.6%) *d* = 0.33
		*P* < 0.001 (82.7–100.0%) *d* = 0.64	*P* < 0.001 (19.8–53.3%) *d* = 0.80	*P* = 0.014 (97.0–100.0%) *d* = 0.57
			*P* = 0.001 (86.3–100.0%) *d* = 1.14	
	Frontal Plane	*P* = 0.008 (92.0 –98.2%) *d* = 0.34	*P* = 0.007 (90.4 –97.0%) *d* = 0.59	None
	Transverse Plane	None	*P* = 0.007 (49.6–55.7%) *d* = 0.61	*P* = 0.003 (48.2 –56.2%) *d* = 0.34
			*P* < 0.001 (82.2–98.6%) *d* = 1.26	
1^st^ MTP Joint	Sagittal Plane	*P* < 0.001 (46.0–95.0%) *d* = 0.65	*P* < 0.001 (43.0–96.5%) *d* = 1.12	*P* < 0.001 (49.4–98.7%) *d* = 0.57
	Frontal Plane	N/A	N/A	N/A
	Transverse Plane	*P* = 0.003 (85.6–96.9%) *d* = 0.82	*P* = 0.006 (31.4–41.6%) *d* = 0.66	*P* = 0.011 (81.3–87.7%) *d* = 0.42
			*P* = 0.003 (82.0–95.4%) *d* = 1.38	

**Notes.**

Loading response = 0–20%; Mid-stance = 20%–50%; Terminal stance = 50%–80%; and Pre-swing = 80%–100%. *P* denotes *p*-value, and *d* denotes Cohen’s *d* effect size.

**Figure 1:**
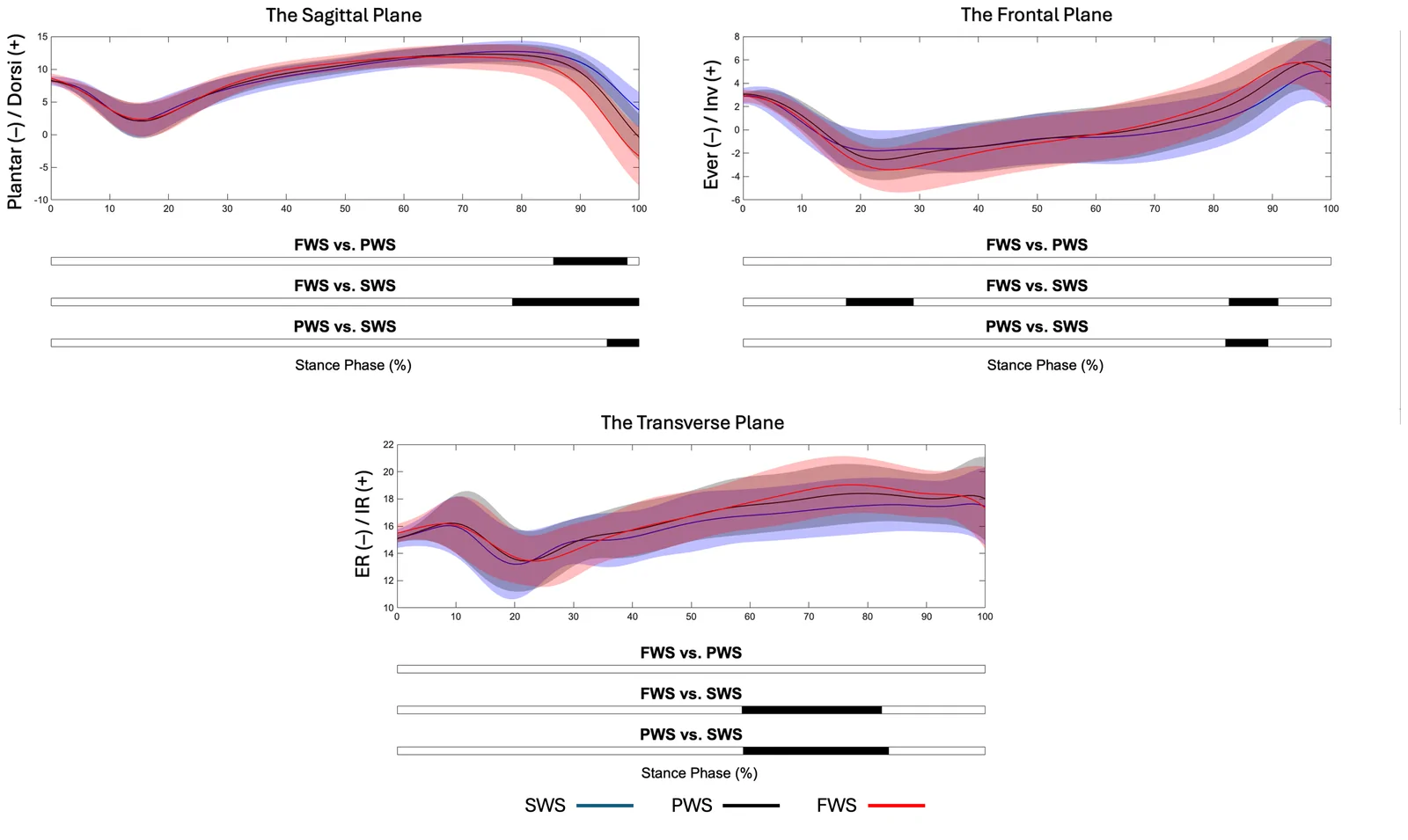
Ankle joint angles (deg) over the stance phase (%). The bold black lines on the *x*-axis represent statistical significance in SPM. SWS, slow walking speed (blue); PWS, preferred walking speed (black); and FWS, fast walking speed (red).

**Figure 2:**
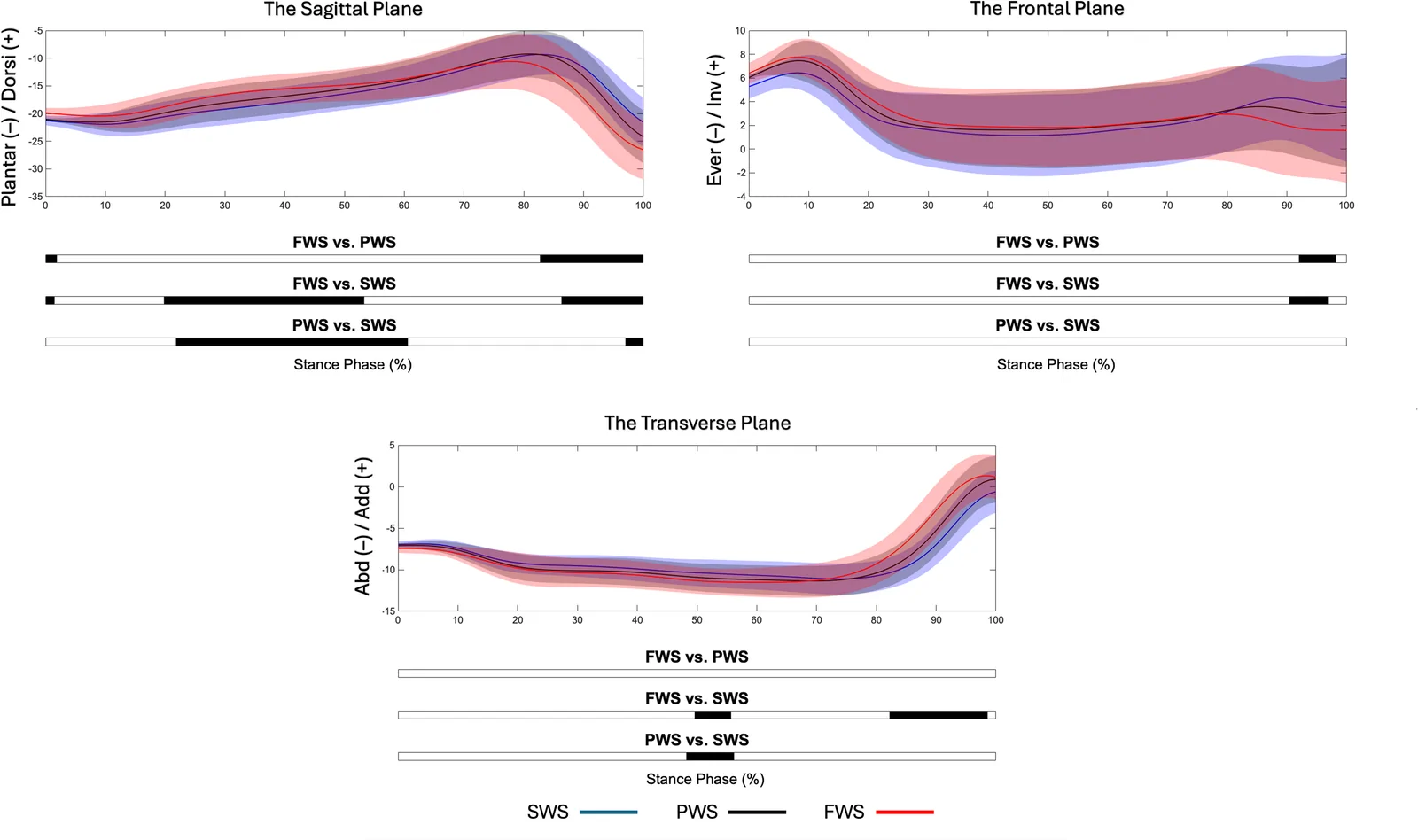
Midfoot joint angles (deg) over the stance phase (%). The bold black lines on the *x*-axis represent statistical significance in SPM. SWS, slow walking speed (blue); PWS, preferred walking speed (black); and FWS, fast walking speed (red).

### The first MTP joint

In the sagittal plane, first MTP dorsiflexion increased progressively with gait speed from terminal stance to pre-swing, being greater in FWS than in PWS (*p* < 0.001; *d* = 0.65) and SWS (*p* < 0.001; *d* = 1.12), and greater in PWS than in SWS (*p* < 0.001; *d* = 0.57) (Table 2, bottom row; Fig. 3, top). In the transverse plane, FWS showed greater first MTP abduction than PWS (*p* = 0.003; *d* = 0.82) and SWS (*p* = 0.003; *d* = 1.38) during pre-swing, while PWS also showed greater abduction than SWS (*p* = 0.011; *d* = 0.42). FWS exhibited less first MTP adduction than SWS during mid-stance (*p* = 0.006; *d* = 0.66), with no significant difference between FWS and PWS (Table 2, bottom row; Fig. 3, bottom).

**Figure 3:**
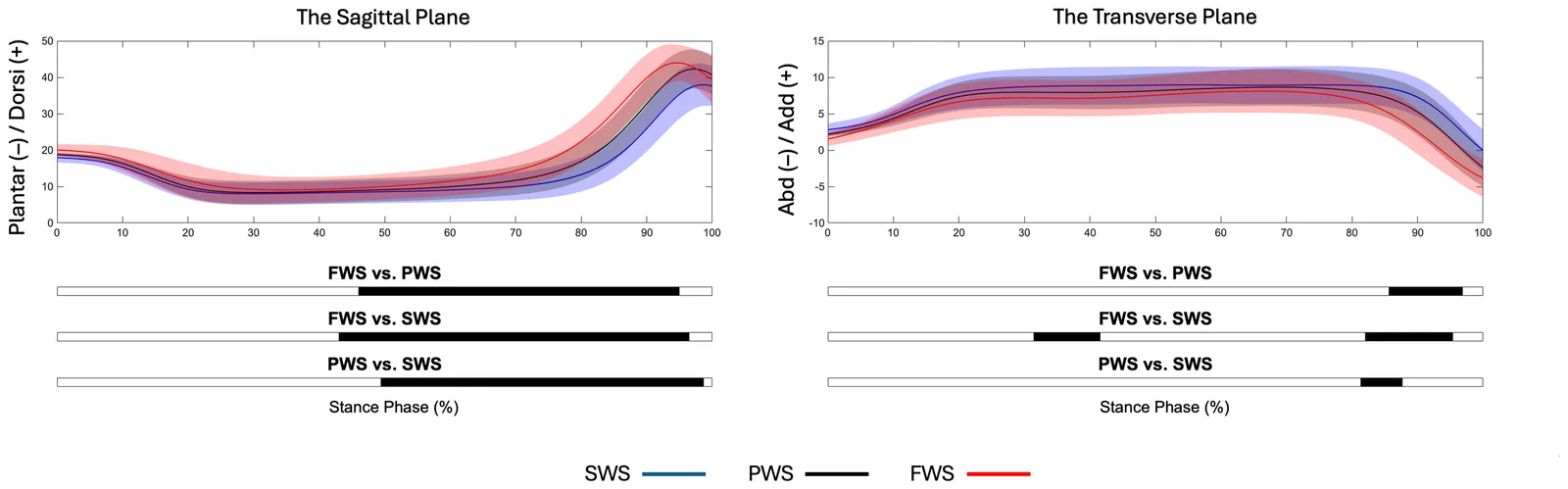
First MTP joint angles (deg) over the stance phase (%). The bold black lines on the *x*-axis represent statistical significance in SPM. SWS, slow walking speed (blue); PWS, preferred walking speed (black); and FWS, fast walking speed (red).

## Discussion

The aim of this study was to investigate the effects of gait speed on the joint kinematics of the multi-segment foot throughout the entire stance phase. We hypothesized that faster walking would increase ankle pronation–abduction–dorsiflexion and midfoot pronation during weight acceptance, whereas it would increase ankle supination–adduction–plantar flexion and midfoot supination during propulsion. In addition, we hypothesized that faster walking would increase first MTP joint dorsiflexion during late stance, consistent with the windlass mechanism. The SPM analyses corroborate our first hypothesis that the effects of gait speed on foot joint kinematics would emerge throughout the stance phase in all three anatomical planes. The statistical analyses also support our second hypothesis in that first MTP joint dorsiflexion was greater at a faster gait speed in late stance. Overall, the multi-segment foot re-organizes its joint kinematics to accommodate changes in gait speed across all three planes.

### Loading response (0–20% of the stance)

During heel strike (approximately 0–3% of the stance) within the loading response, ankle and first MTP joint angles in the sagittal plane remained consistent across the different gait speeds. The consistent ankle joint angles in the sagittal plane correspond to the similar peak ankle plantar flexion observed across different walking speeds during the first rocker phase—a discrete time window from heel strike to the first local maximum plantar flexion of the ankle joint (Tulchin et al., 2009). In contrast, the reduced midfoot plantar flexion (*i.e.,* greater dorsiflexion) observed at FWS during heel strike (Fig. 2, top) may reflect the higher impact forces at FWS inducing greater posterior compression of the hindfoot, which could passively extend the midfoot joint. However, unlike in the present study, Sun et al. (2018) reported consistent midfoot dorsiflexion across gait speeds, along with greater peak ankle dorsiflexion and eversion, and reduced peak midfoot supination and adduction during fast walking. This coupling pattern reflects foot pronation relevant to arch collapse, which typically occurs immediately after heel strike. This discrepancy between studies likely arises from methodological differences in defining heel strike. In the present study, heel strike was defined as the first local minimum of the vertical heel marker trajectory, whereas Sun et al. (2018) defined it as the moment when the vertical ground reaction force exceeded 30 N. Since gait speed is closely associated with impact forces, defining heel strike based on a force threshold may result in its detection occurring slightly later than the actual event, particularly at slower speeds.

### Mid-stance (20–50% of the stance)

From late loading response (beginning around 20% of stance), fast walking speed was associated with greater ankle eversion compared to both slow walking speed and preferred walking speed (Fig. 1, middle), suggesting increased foot arch deformation. In contrast, increased midfoot dorsiflexion at fast walking speed was observed only when compared to slow walking speed (approximately 17–30% of the stance) but not to preferred walking speed (Fig. 2, top). These findings suggest that arch deformation during faster walking is primarily driven by increased ankle eversion, with an additional contribution from midfoot dorsiflexion relative to slower walking speeds. Meanwhile, no differences were observed between preferred walking speed and slow walking speed for either ankle eversion or midfoot dorsiflexion, which is consistent with a previous study by Sun et al. (2018). However, Sun et al. (2018) reported greater peak ankle external rotation at faster walking speeds during mid-stance, which may reflect the sensitivity of the results to the foot model used. Concurrently, first MTP adduction decreased during mid-stance (approximately 31–41% of the stance, Fig. 3, bottom), followed by increased first MTP dorsiflexion (beginning around 43% of the stance, Fig. 3, top) and midfoot abduction (approximately 48–56% of the stance, Fig. 2, bottom) during faster walking. This sequence of changes at faster speeds suggests increased deformation of the foot arch, leading to greater pressure on the hallux. The resultant arch collapse may contribute to the absorption of mechanical energy by potentially prolonging the period of conservative impulse (Whittle, 1999) and may be associated with the storage of additional energy through the conversion of mechanical forces into strain energy within the plantar soft tissues (Ker et al., 1987).

### Terminal stance (50–80% of the stance)

Starting from mid-stance, the increased midfoot dorsiflexion observed during faster walking (approximately 48–56% of the stance, Fig. 2, top) progresses into greater midfoot abduction in early terminal stance, accompanied by a substantial increase in hallux dorsiflexion that continues through terminal stance and into pre-swing. Around 60% of stance, this sequence is followed by the onset of ankle internal rotation, which also persists through terminal stance and into early pre-swing. The emergent multiplanar behavior across the foot joints may reflect a forward–medial shift of the center of pressure, directing body weight toward the first ray of the foot. This shift in force emerging in early stance may facilitate more effective propulsion during toe-off, as the longer length of the hallux and the presence of the sesamoid bones beneath the first metatarsal head provide a relatively longer moment arm and larger radius of rotation, respectively (Hicks, 1953; Huson, 1991). These structural features are thought to possibly increase foot stiffness, thereby potentially enhancing the transmission of energy from the proximal to the distal foot *via* strain energy storage in the plantar soft tissues (Ker et al., 1987). This shift, however, was not reported by Sun et al. (2018), who observed consistent peak ankle internal rotation across gait speeds, but greater peak midfoot abduction at fast walking speeds, which may suggest an outward shift of the center of pressure during terminal stance.

### Pre-swing (80–100% of the stance)

Ankle inversion, in combination with plantar flexion, is associated with midtarsal locking (Bojsen-Møller, 1979). This may explain why at faster speed greater ankle plantar flexion (around 85–95% of stance, Fig. 1, top) and inversion (around 80–90% of stance, Fig. 1, middle) appear to enhance propulsive force during toe-off by stabilizing the foot and enabling increased midfoot plantar flexion (around 90–100% of stance, Fig. 2, top) and first MTP dorsiflexion (around 80–95% of stance, Fig. 3, top). Moreover, reduced midfoot inversion (around 90–95% of the stance, Fig. 2, middle) and greater midfoot adduction (around 83–97% of the stance, Fig. 2, bottom) at FWS indicate a pronation twist at the forefoot, leading to greater arch elevation and tension of the foot (Hicks, 1953). This, in turn, may cause the hallux to be passively abducted against the supporting surface (around 85–93% of the stance, Fig. 3, bottom). This series of foot motion patterns represents foot supination, marked by increased arch height and a stiffer foot structure that theoretically promotes propulsive function (Hicks, 1954). This resulting foot pattern aligns with previous studies reporting greater peak ankle and midfoot plantar flexion (Tulchin et al., 2009) and peak hallux dorsiflexion (Dubbeldam et al., 2010). Conversely, Sun et al. (2018) reported consistent peak ankle plantar flexion and even reduced peak midfoot plantar flexion and first MTP dorsiflexion across walking speeds. Although these peak joint angles are expected to occur during pre-swing, at least in healthy young adults, their precise timing remains unclear, as discrete joint kinematics do not account for temporal factors. This limitation may give rise to inconsistencies among previous studies (Tulchin et al., 2009; Dubbeldam et al., 2010; Sun et al., 2018).

### Limitations

This study has some limitations. The multi-segment foot model we applied is different from the model used by previous studies (Tulchin et al., 2009; Sun et al., 2018). However, we chose our foot model developed by Bruening, Cooney & Buczek (2012) because the foot model showed higher inter- and intra-repeatability, a key factor when modelling small body segments of the foot. This inter-trial repeatability is supported by our kinematic curves, which closely resemble those reported by Bruening, Cooney & Buczek (2012). In addition, we did not specifically screen the foot types of the participants (*e.g.*, low, normal, and high arch) by quantifying foot-arch height. However, we assumed that our participants had normal arch height based on an online pre-screening questionnaire and a visual assessment of foot posture conducted by a certified clinician prior to the experiment. We excluded those with conditions affecting walking, for instance, due to foot deformities (*e.g.*, flat foot) and any known neuromuscular diseases or injuries *via* the questionnaire.

The tested walking speed in the fast condition was approximately 1.4 m⋅s^−1^, which is close to typical overground normal walking speed (∼1.4 m⋅s^−1^). However, since the speed conditions were defined relative to each participant’s preferred walking speed (*i.e.,* 70% and 130%), the absolute walking speeds varied across individuals. Therefore, although the mean speed of the fast condition was close to 1.4 m⋅s^−1^, individual walking conditions may have resulted in altered foot joint kinematics due to differences in mechanical demands.

## Conclusion

This study identified adaptive foot kinematics in response to walking speed, manifested as multiplanar changes across proximal-to-distal ankle–foot joints throughout the stance phase. Such systematic changes in joint kinematics underscore the importance of continuous analyses, which offer novel insights by capturing spatiotemporal information that may be overlooked by discrete measures. Further investigation is warranted to characterize the influence of walking speed on foot energy–time trajectories and to determine how these kinematic adaptations can facilitate effective shock attenuation and propulsion and contribute to whole-body gait efficiency, ultimately guiding evidence-based rehabilitation approaches and the design of supportive interventions such as orthotics, prosthetics, and sports taping.

##  Supplemental Information

10.7717/peerj.21787/supp-1 Appendix A Reflective Markers to create and track body segments

10.7717/peerj.21787/supp-2 Appendix B Multi-segment foot model adapted from Bruening, Cooney & Buczek (2012)

10.7717/peerj.21787/supp-3 Data S1 Foot Joint Kinematic Data raw data of ankle (9 files), midfoot (9 files), and 1st MTP joint kinematics (6 files). Each CSV file contains 12 rows (representing the number of subjects) and 101 columns (representing the number of data points across the stance phase). In the CSV file titles, 70, 100, and 130 indicate the slow, preferred, and fast walking speed conditions, respectively. In addition, S, F, and H in the CSV file titles represent the sagittal, frontal, and horizontal planes, respectively.

## Additional Information and Declarations

### Data Availability

The following information was supplied regarding data availability: The data is available in the Supplemental Files.
