# Supplementary material for: Gait speed and multi-segment foot joint kinematics throughout the entire stance phase using statistical parametric mapping
Source: PeerJ. 2026 Oct 21;14:e21787. doi: 10.7717/peerj.21787 (PMC13695423; doi:10.7717/peerj.21787)
Supplement: Supplementary file 1 [file peerj-14-21787-s001.pdf]

Appendix A: Reflective Markers to create and track body segments (**Bruening et al., 2012**)

Segment	Plane markers	Tracking markers
R & L one rigid foot	ACC	Peroneal tubercle
	Apex of calcaneal tuberosity	Apex of calcaneal tuberosity
	Superior apex of calcaneus	Superior apex of calcaneus
R & L Hindfoot	Apex of calcaneal tuberosity	Peroneal tubercle
	MTC	Apex of calcaneal tuberosity
	Superior apex of calcaneus	Superior apex of calcaneus
R & L Forefoot	MTC	1st metatarsal head
	FF_Dist	2nd metatarsal head
	2nd metatarsal head	5th metatarsal base
R & L Hallux	MPC	1st metatarsal head
	HX_Dist	Hallux distal phalange
	1st metatarsal head	H1_plane
R & L Shank	Lateral knee epicondyle	Shank cluster (non-collinear four markers on a rigid rectangular plate located on the lateral mid-surface of the shank)
	Medial knee epicondyle	
	Lateral ankle malleolus	
	Medial ankle malleolus	

R & L Hip	Hip[Ω]<br>Lateral knee epicondyle<br>Medial knee epicondyle	Thigh cluster (non-collinear four markers on a rigid rectangular plate located on the lateral mid-surface of the thigh)
Pelvis	R & L anterior superior iliac spine<br>R & L posterior superior iliac spine	Pelvic cluster (non-collinear three markers on a rigid circular plate on the sacrum)
Trunk	Pelvis_C<br>R & L acromion processes	Trunk cluster (non-collinear three markers on a rigid circular plate on the manubrium)
R & L upper arm	Acromion process<br>Lateral humeral epicondyle<br>Medial humeral epicondyle	Anterior-proximal aspect of the shoulder<br>Posterior-proximal aspect of the shoulder<br>Lateral-mid aspect of the humerus
R & L forearm	EBC<br>Radial styloid process<br>Ulnar styloid process	Lateral humeral epicondyle<br>Lateral-mid aspect of the forearm<br>Radial styloid process
Head	Head_C<br>Chin<br>R forehead	R & L foreheads<br>R & L rear heads

Ω = Based on the method of Davis et al., 1991 (**Davis et al., 1991**)

ABBREVIATIONS:

ACC = Midpoint between lateral ankle and medial ankle malleoli

MTC = Midpoint between the navicular and cuboid markers

MPC = 1/2 vertical projection of the 1st metatarsal head marker to the ground.

FF_Dist = 1/2 vertical projection of the 2nd metatarsal head marker to the ground

HX_Dist = 1/2 vertical projection of the hallux marker to the ground

H1_Plane = Coordinated marker with the mediolateral plane of the forefoot segment

Pelvis_C = Midpoint among the four pelvic markers

EBC = Midpoint between lateral and medial humeral epicondyles

Head_C = Midpoint between the four non-collinear head markers

Chin =1/2 vertical projection between Head_C and the midpoint between 7th cervical marker and the sternum marker on the manubrium

REFERENCE

1. Bruening, D. A., Cooney, K. M., & Buczek, F. L. (2012). Analysis of a kinetic multi-segment foot model. Part I: Model repeatability and kinematic validity. *Gait & posture*, *35*(4), 529-534.
2. Davis RB, Ounpuu S, Tyburski D, Gage JR. (1991) “A Gait Analysis Data Collection and Reduction Technique.” Human Movement Science 10: 575-587.
