# Supplementary material for: Gait speed and multi-segment foot joint kinematics throughout the entire stance phase using statistical parametric mapping
Source: PeerJ. 2026 Oct 21;14:e21787. doi: 10.7717/peerj.21787 (PMC13695423; doi:10.7717/peerj.21787)
Supplement: Supplementary file 2 [file peerj-14-21787-s002.pdf]

Supplemental figure: Multi-segment foot model adapted from Bruening et al. (2012).

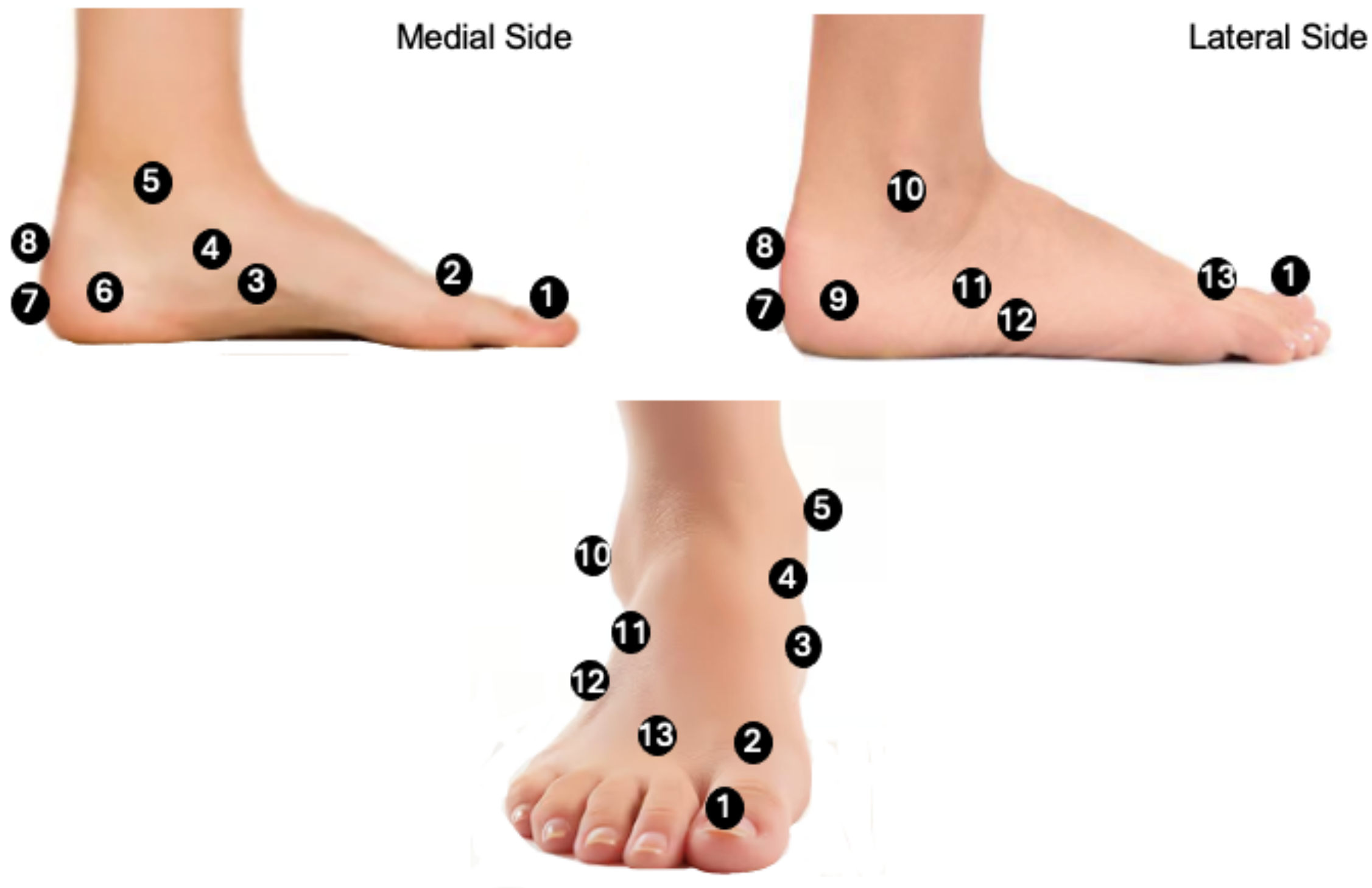

1 = Hallux

2 = First metatarsophalangeal joint

3 = Base of the first metatarsal

4 = Navicular tuberosity

5 = Medial malleolus

6 = Medial calcaneus

7 = Posterior calcaneus

8 = Superior calcaneus

9 = Lateral calcaneus

10 = Lateral malleolus

11 = Cuboid

12 = Base of the fifth metatarsal

13 = Marker positioned between the second and third metatarsophalangeal joints

REFERENCE

1. Bruening, D. A., Cooney, K. M., & Buczek, F. L. (2012). Analysis of a kinetic multi-segment foot model. Part I: Model repeatability and kinematic validity. *Gait & posture*, *35*(4), 529-534.
